# Improved Oxidation Resistance of Graphite Block by Introducing Curing Process of Phenolic Resin

**DOI:** 10.3390/ma16093543

**Published:** 2023-05-05

**Authors:** Jong-Hwan Ko, Sang-Hye Lee, Jae-Seung Roh

**Affiliations:** School of Materials Science and Engineering, Kumoh National Institute of Technology, Gumi 39177, Republic of Korea

**Keywords:** phenolic resin, curing, carbonization, graphite block, oxidation resistance, graphitization

## Abstract

The purpose of this study is to improve the oxidation resistance of graphite blocks after graphitization at 2800 °C by introducing a curing process of phenolic resin, used as a binder to control the pore size. Using the methylene index obtained from FTIR, the curing temperature was set to 150 °C, the temperature at which cross-linking most highly occurs. Graphite blocks that had undergone curing, and were carbonized with a slow heating rate, showed increased mechanical and electrical properties. Microstructural observation confirmed that the curing process inhibited the formation of large pores in the graphite block. Therefore, the cured graphite block showed better oxidation resistance in air than a non-cured graphite block. Oxidation of the graphite block was caused by pores created by pyrolysis of the phenolic resin binder, which acted as active sites.

## 1. Introduction

Isotropic graphite blocks are manufactured by cold isostatic pressing (CIP) and have the same properties regardless of orientation [1]. They are made through a series of processes, including raw material grinding, mixing, kneading, molding, carbonization, impregnation, re-carbonization, and graphitization; a purification step may be added when necessary [2]. In this process, petroleum and coal-based isotropic pitch coke are used as filler, and organic materials such as pitch and phenolic resin as binders [1,3].

Phenolic resin, a binder in graphite block manufacturing, is used primarily for oxide-carbon and other refractory materials. Since its beginning in a patent issued to Leo Baekeland in 1907, phenolic resin has been widely used as a thermosetting resin in various fields, from construction to electronics and aerospace [4]. Final products made from phenolic resin offer high carbonization yield, adhesive strength, and strength; they are also less hazardous than pitch binders [5]. However, one disadvantage of phenolic resin is that its use can cause cracks to form easily inside graphite blocks due to the high degree of shrinkage during heat treatment [6].

Artificial graphite has the disadvantage of undergoing oxidation reactions in atmospheres containing O_2_, CO_2_, and H_2_O; it generally oxidizes at temperatures above 450 °C in air [7,8]. Such reactions cause deterioration of mechanical strength and electrical properties in materials used in fields involving high-temperature technology, such as nuclear power and refractories [7,9]. As such, many researchers have added materials and applied coatings to prevent the oxidation of graphite blocks [10,11,12]. Zhao, et al., reported that nickel additives can improve the oxidation resistance of phenolic resin binder and enhance high-temperature properties [13]. It is difficult to find studies showing improvements in oxidation resistance through heat treatment of binder phenolic resin during graphite block manufacturing.

The oxidation reactions of graphite occur primarily at active sites located at the ends of the basal planes of graphite crystals, which are found on pore walls [14]. During the carbonization process in graphite block manufacturing, organic matter from phenolic resin is pyrolyzed into carbon, while the remaining volatile matter is released into the atmosphere to form open pores in the graphite blocks [15,16,17]. The pores (porous structures) formed at this time work as active sites during oxidation [18]. This demonstrates the close relationship between the oxidation of carbon materials and a porous structure, highlighting the importance of improving oxidation resistance to minimize open pores.

Our research team, based on extensive experience in manufacturing graphite blocks, has been exploring ways to improve the oxidation resistance of binder phenolic resins without additives [19,20]. One promising method is to inhibit the formation and reduce the size of open pores in the binder phenolic resins. Glassy carbon can be obtained by slowly curing and carbonizing phenolic resin at a slow heating rate [21,22,23]. While glassy carbon (1.30~1.55 g/cm^3^) has a lower density than commercial graphite blocks (1.68~1.91 g/cm^3^), it has very low gas and liquid permeability because there are hardly any structurally open pores [24,25]. Glassy carbon with low open porosity is used in biomaterials that require non-reactive environments, such as health valves and scaffolds for tissue regeneration [26,27,28].

We introduced a curing process for phenolic resin similar to that used in glassy carbon manufacturing and minimized the open porosity of graphite blocks to improve their oxidation resistance. The decrease in open porosity was expected to enhance flexural strength and electrical conductivity.

The aim of this study was to fabricate graphite blocks with improved oxidation resistance by modifying the curing and carbonization processes of the binder phenolic resin. First, the curing conditions were determined by examining the crystallinity and microstructure (porosity) of the phenolic resin according to the curing process; the curing conditions for the phenolic resin were applied to produce artificial graphite. Quicker production was achieved at a faster carbonization rate. Finally, we compared the physical properties of the resulting graphite blocks to those of existing graphite blocks.

## 2. Experimental Procedure

To determine the curing temperature that minimizes porosity during graphite block fabrication, properties of samples obtained by curing phenolic resins at low temperatures followed by carbonization were analyzed.

Cured phenolic resins prepared at different curing temperatures were analyzed by FTIR to determine the degree of cross-linking, and the curing temperature with the most active cross-linking was determined. Cross-linking is a process in which aromatic rings of phenolic resins are connected while oxygen and hydrogen-containing functional groups are released in the form of H_2_O [29]. Therefore, it was presumed that cross-linking would reduce the amount of volatile gas released during the carbonization process.

TGA analysis was used to compare the thermal properties of cured and non-cured phenolic resins; microstructures of carbonized phenolic resins were observed for comparison of pore size.

**Figure 1:**
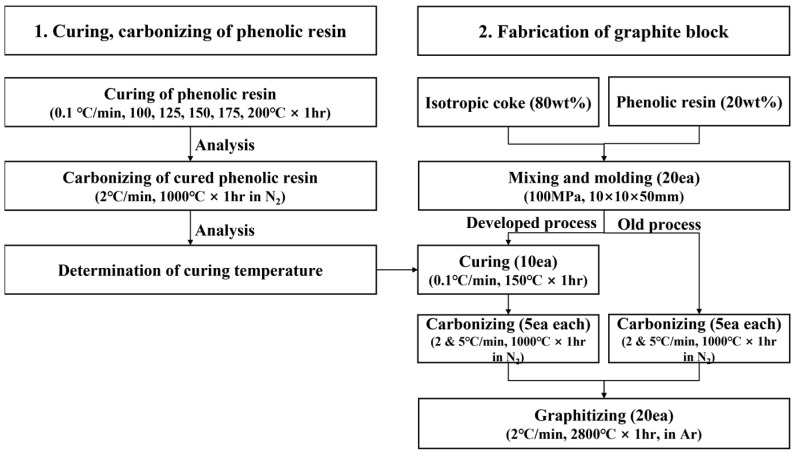
The flowchart of experiments.

Graphite blocks were then fabricated using various heat treatment process variables, and physical properties and oxidation resistance were compared. Figure 1 presents the overall experimental procedures.

### 2.1. Curing, Carbonizing, and Analysis of Phenolic Resin

#### 2.1.1. Curing and Carbonizing of Phenolic Resin

A solution mixture was prepared by mixing phenolic resin (CB-8081, Kangnam Chemical Co., Ltd., Seoul, Republic of Korea) and 99.5% pure isopropyl alcohol (IPA, Duksan General Science, Seoul, Republic of Korea) at a weight ratio of 5:5. The mixture was heated at a rate of 0.1 °C/min in a box furnace and, after reaching the target temperature, cured for one hour. Phenolic resin is known to undergo cross-linking at temperatures between 100 °C and 190 °C [30,31]. As such, the curing temperatures were set to 100, 125, 150, 175, and 200 °C. The cured phenolic resin was carbonized in N_2_ atmosphere at a heating rate of 2 °C/min up to 1000 °C; this final temperature was then maintained for one hour. The cured and carbonized phenolic resin is hereinafter called “cured and carbonized phenol”.

#### 2.1.2. Methylene Index from FTIR

To understand the curing mechanism of cured phenol at various curing temperatures, Fourier transform infrared spectroscopy (FTIR) analysis was performed. The ATR mode (INVENIO X, Bruker, Billerica, MA, USA) was employed, and the wave number range was 900 to 3600 cm^−1^.

Hu, et al., and other researchers applied FTIR to examine the curing mechanism of phenolic resin. They calculated the methylene index (p-p) using the ratio of the adsorption intensity (*A*_1610_) of C=C, representing the aromatic ring of phenol, to the adsorption intensity of CH_2,_ representing the methylene bond [32,33]. Here, a higher methylene index indicates a higher degree of cross-linking. The methylene index is calculated as follows.

Methylene index (p-p) = *A*_(1480)_/*A*_(1610)_ (1)

#### 2.1.3. Weight Change with Regard to Temperature from TG-DTG

Thermal gravimetric analysis (TGA, Auto TGA Q500, TA Instruments, New Castle, DE, USA) was carried out to examine the weight changes of phenolic resin with and without curing at varying temperatures. The samples were heated at 2 °C/min up to 900 °C in an N_2_ atmosphere, and the weight changes were measured. In addition, the carbonization yield and mass change points were also determined.

#### 2.1.4. Observation of Pores

Microstructures were observed to compare the pore size of carbonized phenol with and without curing. The samples were polished to 0.25 μm using diamond paste and observed with a FESEM (JSM-6500F, JEOL, Tokyo, Japan).

### 2.2. Fabrication and Analysis of Graphite Block

#### 2.2.1. Fabrication of Graphite Block

Isotropic coke powder with an average particle size of 7.5 μm was used as a filler; phenolic resin was used as a binder. The isotropic coke and phenolic resin were mixed at a weight ratio of 8:2. Uniaxial pressure of 100 MPa was applied to prepare 20 samples measuring 10 × 10 × 50 mm. Ten out of the 20 samples were cured and carbonized, while the remaining 10 were carbonized without curing. The curing conditions are provided in Section 2.1.1. Carbonization was performed by heating in N_2_ atmosphere up to 1000 °C and maintaining the temperature for one hour.

A previous study by our team found that the properties of graphite blocks increased when heating rate during carbonization was adjusted to below 3 °C/min. Graphite blocks prepared at heating rates higher than 5 °C/min had large pores and cracks in their microstructures and exhibited low flexural strength and high electrical resistivity [20].

Accordingly, this study performed carbonization at a heating rate of 2 °C/min, below the previously reported heating rate of 3 °C/min [20]. To compensate for the increased manufacturing time due to the curing process and to improve the production speed, a faster heating rate of 5 °C/min was applied. The carbonized blocks were graphitized by maintaining them at a temperature of 2800 °C in Ar atmosphere for one hour.

In this study, graphite block samples subject to curing were represented by “C”, the heating rate during carbonization by “HRn”, and graphitization by “G”. Table 1 shows naming of graphite blocks in this study. 

#### 2.2.2. Bulk Density and Porosity of Graphite Block

The bulk density and porosity of the graphite blocks were calculated using the Archimedes method (ISO 18754) by measuring the underwater weight, saturated weight, and dry weight of five specimens under varying conditions. The calculated average values are displayed in the graphs, together with dispersion (identical for subsequent tests).

Bulk density (g/cm^3^) = Dry weight/(saturated weight-underwater weight) 
Porosity (%) = {(Saturated weight − dry weight)/(Saturated weight − underwater weight)} × 100(2)

#### 2.2.3. Electrical Resistivity of Graphite Block

The electrical resistivity of the graphite blocks was measured using the voltage drop method according to ASTM C 611 for the five samples under varying conditions. The voltage drop between voltage terminals, the cross-sectional area of samples, the current, and the distance between terminals were measured; the electrical resistivity was calculated as follows:

⍴ = eS/il(3)

where, ⍴ is electrical resistivity (Ωcm); e is voltage drops between voltage terminals (V); S is cross-sectional area of samples (cm^2^); i is current (A); l is distance between voltage terminals (cm).

#### 2.2.4. Flexural Strength of Graphite Block

The flexural strength of the graphite blocks was measured using a three-point bending test of ASTM D 7972 for the five samples under varying conditions. A universal testing machine (Quasar 100, Galdabini, Cardano al Campo, Italy) was used; measurements were obtained with a distance of 40 mm between the two lower points and a crosshead speed of 0.5 mm/min.

S_b_ = 3WI/2bt^2^ (4)

where, S_b_ is the flexural strength (N/cm^2^); I is distance between points (cm); W is maximum load (N); b is sample width (cm); t is sample thickness (cm).

#### 2.2.5. Pore Analysis of Graphite Blocks

Microstructure observations were performed to compare the pore size of the graphite blocks with and without curing. The graphite blocks were polished to 0.25 μm using diamond paste and observed under an optical microscope (OM, ECLIPSE LV150, Nikon, Tokyo, Japan) at a magnification of ×200. FESEM was used to observe the samples at magnifications of ×100 and ×500.

Pore size distribution of graphite blocks was performed using a porosimeter (Auto pore V, Micromeritics). Based on the Washburn equation, mercury, which is non-wetting for almost all materials, was infiltrated into graphite blocks at pressures ranging from 0 to 60,000 psi. Graphite blocks were prepared to 10 × 10 × 10 mm to fit inside the tube, and pore sizes were measured from 3 to 360,000 nm.

#### 2.2.6. Oxidation Resistance Test

To compare the oxidation resistance of graphite blocks in relation to the heat treatment process variables, weight changes were examined after oxidization using air gas at 650 °C. The blocks were placed in a tube furnace at 650 °C in N_2_ atmosphere and maintained for 15 min. The atmosphere was switched to air gas with a flow of 100 mL, maintained for two hours, and then oxidized. After oxidization, the atmosphere was switched back to N_2_, and the tube furnace was cooled to 100 °C before collecting the graphite blocks. The weight changes of the blocks before and after oxidization were measured, and FESEM was used in the microstructural analysis.

## 3. Results and Discussion

### 3.1. Analysis of Cured and Carbonized Phenol

#### 3.1.1. Methylene Index from FTIR

**Figure 2:**
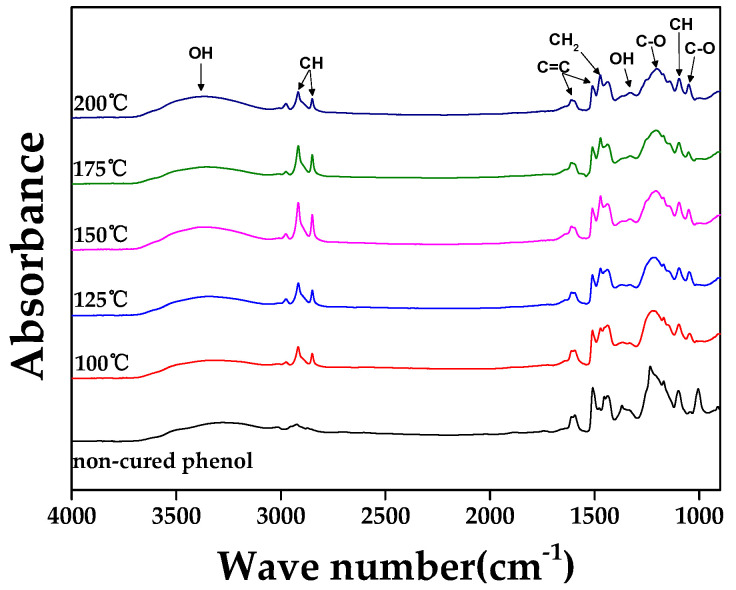
FTIR spectra of phenolic resin and cured phenol in relation to curing temperature.

Figure 2 shows the FTIR spectra of cured phenol in relation to curing temperature. Table 2 presents observed wave numbers (cm^−1^) and functional groups. OH peaks were observed at 3272–3364 cm^−1^, aliphatic CH at 2923~2916 cm^−1^, aromatic C=C at 1594–1610 cm^−1^ and 1508–1509 cm^−1^, aliphatic CH_2_ at 1438~1472 cm^−1^, C-O at 1206~1234 cm^−1^ and 1005–1050 cm^−1^, and aliphatic CH at 1095–1099 cm^−1^ [34,35].

Phenolic resins undergo cross-linking, pyrolysis, and condensation during curing. First, the OH^−^ of the methylol group (CH_2_OH) reacts with H^+^ and is volatilized into H_2_O form; the remaining CH_2_ is cross-linked with phenolic monomers, increasing the molecular weight. Finally, the low molecular weight of phenolic resin is released through pyrolysis [29].

The pyrolysis of phenolic resin is typically interpreted in three temperature ranges: low (<200 °C), medium (200~600 °C), and high (>600 °C). Moisture is released as condensation in the low-temperature range, as CO_2_, CH_4_, and CO in the medium range, and as H_2_ in the high range. Gas release occurs rapidly in the medium-temperature range, resulting in the formation of large pores [20,36].

During the curing of phenolic resin, -CH_2_, -OH, and C-O, which contribute to the production of gases (CH_4_ and CO_2_) in the medium-temperature range, may be released early at low temperatures. This can allow graphite blocks to have smaller pores. Figure 2 shows the increased intensity of CH_2_ peaks due to more active cross-linking up to 150 °C, followed by slight decreases at 175 and 200 °C. For all cured resins, there was a decrease in C-O peak intensity due to moisture release and a decrease in C=C peak intensity due to the pyrolysis of aromatic rings with low molecular weights.

**Figure 3:**
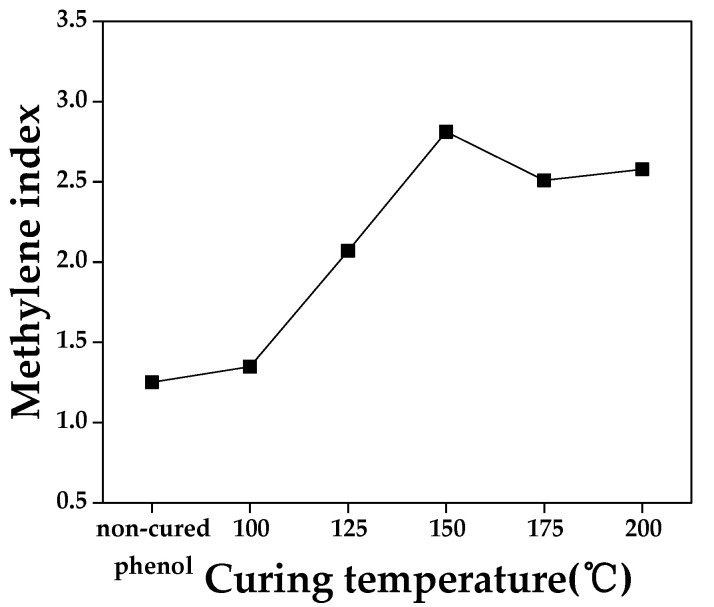
Methylene index in relation to curing temperature of phenolic resin.

Figure 3 shows the methylene index (p-p) in relation to the curing temperature of phenolic resin. The methylene index (p-p) reached its maximum value at 150 °C, which is consistent with the increase in the intensity of the CH_2_ peak up to 150 °C and the decrease in C=C intensity after curing. The curing temperature for graphite block fabrication was thus set at 150 °C to maximize cross-linking.

#### 3.1.2. Weight Change with Regard to Temperature from TG-DTG

**Figure 4:**
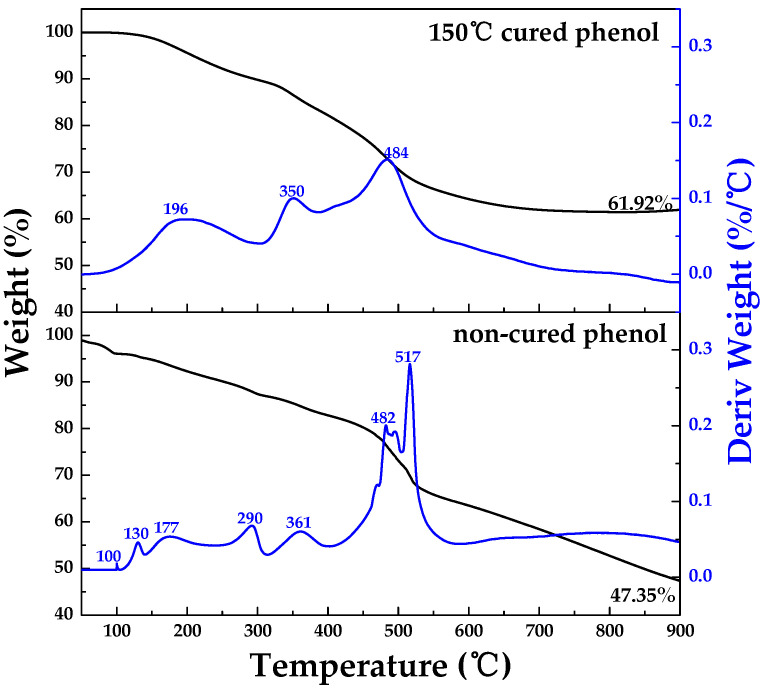
TG-DTG of phenolic resin in relation to curing status.

Figure 4 shows the TG-DTG analysis results of phenolic resin in relation to curing. The carbonization yields of non-cured phenol and 150 °C-cured phenol at 900 °C were 47.35% and 61.92%, respectively.

As can be seen in the DTG graph, non-cured phenol had mass loss peaks at 100, 130, 177, 290, 361, 482, 496, and 517 °C. A sharp maximum mass loss peak was observed in the range of 482–517 °C. On the other hand, 150 °C-cured phenol had mass loss peaks at 196, 350, and 484 °C; the maximum mass loss peak appeared in broad form at 484 °C. This can be interpreted as showing a reduction in CO_2_ and CH_4_ gas released at around 500 °C due to the pre-release of OH^−^ and H^+^ groups through cross-linking during curing, as shown in the FTIR results.

#### 3.1.3. Pore Observation of Carbonized Phenol

**Figure 5:**
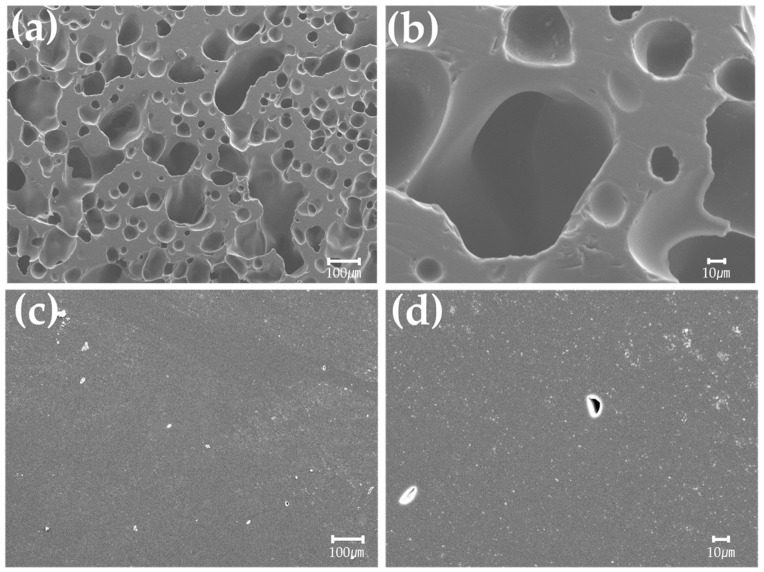
Microstructure of carbonized (**a**) non-cured phenol (×100), (**b**) non-cured phenol (×500), (**c**) 150 °C-cured phenol (×100), and (**d**) 150 °C-cured phenol (×500).

Figure 5 shows SEM images of phenolic resin after carbonization in relation to curing status. Non-cured phenol exhibits very large pores that can be easily observed at ×100 magnification, with some very large pores exceeding 100 µm (Figure 5a,b). On the other hand, 150 °C-cured phenol did not have the large pores that were observed in non-cured phenol (Figure 5c,d) because large pores formed irregularly in non-cured phenol due to the rapid release of volatiles at around 500 °C. The formation of large pores was inhibited in 150 °C-cured phenol because fewer volatiles were released at low temperatures during curing.

### 3.2. Analysis of Graphite Block

#### 3.2.1. Bulk density and porosity of graphite block

**Figure 6:**
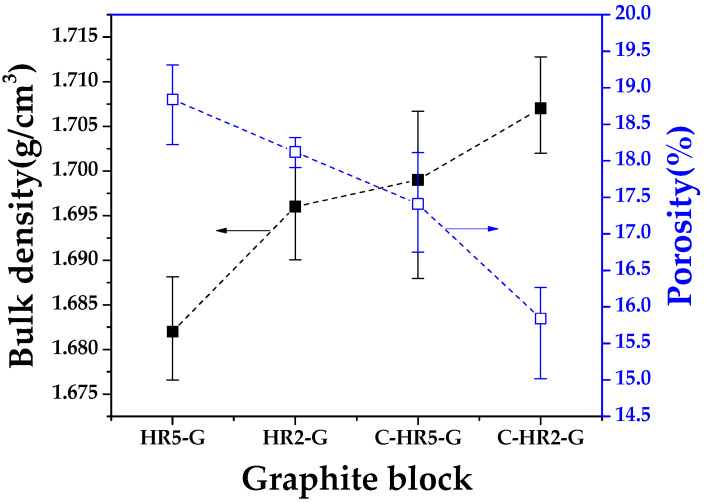
Bulk density and porosity of graphite block in relation to curing and carbonization conditions.

Figure 6 shows the bulk density and porosity of the graphite blocks in relation to curing and carbonization conditions. C-HR2-G showed the best values, with a bulk density of 1.707 g/cm^3^ and porosity of 15.8%.

Our research team reported that a rapid heating rate during carbonization when fabricating graphite blocks with a phenolic resin binder led to low bulk density and high porosity [20]. However, a comparison of C-HR5-G and HR2-G revealed that C-HR5-G has a higher bulk density and lower porosity. This demonstrates that the curing process of phenolic resin is more effective for pore size control than is heating rate during carbonization. This result is expected to be useful in application to actual manufacturing sites, where product sizes are large, and production speeds are slow.

#### 3.2.2. Electrical Resistivity and Flexural Strength of Graphite Block

**Figure 7:**
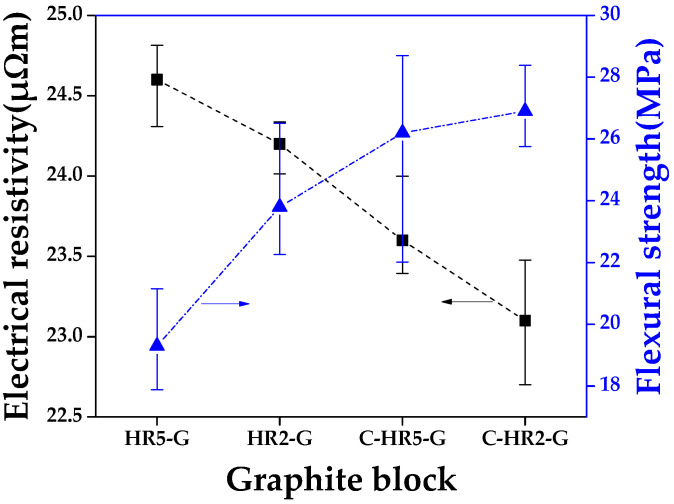
Flexural strength of graphite blocks in relation to curing and carbonization conditions.

Figure 7 shows the electrical resistivity and flexural strength of the graphite blocks in relation to curing and carbonization conditions. C-HR2-G had the lowest electrical resistivity of 23.1 μΩm and the highest flexural strength of 26.9 MPa.

Stress concentrates at the pores of the graphite blocks; pore size thus affects mechanical strength [37]. As can be seen in the above porosity results, low electrical resistivity and high flexural strength can result from the inhibited formation of large pores during curing.

#### 3.2.3. Pore Observation of Graphite Block

**Figure 8:**
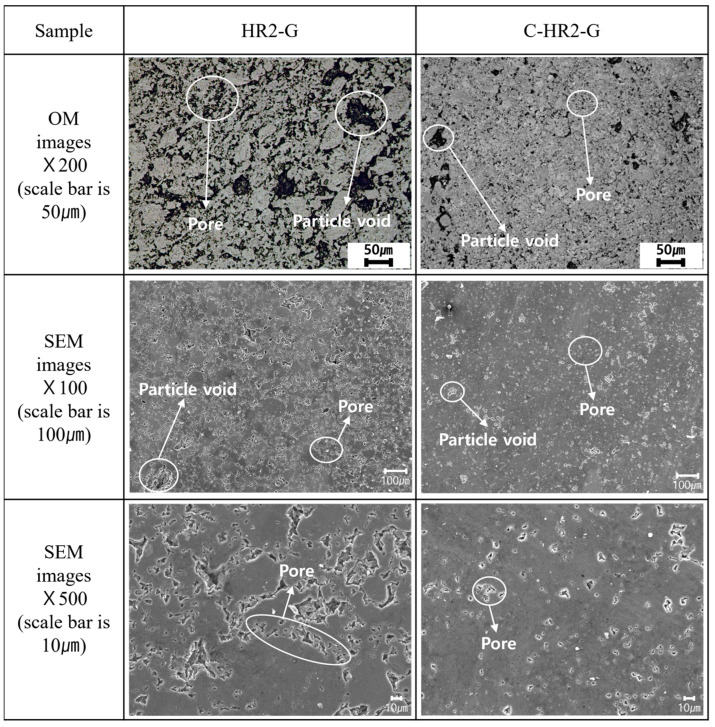
Microstructures of HR2-G and C-HR2-G.

Figure 8 shows the microstructures of HR2-G and C-HR2-G. Both samples had voids ranging from 10 to 50 μm in size, appearing as black spots in the images. The average particle size of the filler used to fabricate the graphite blocks was 7.5 μm; pores between fillers of this size would be even smaller. As such, observed voids are probably particle voids formed during grinding, during which particles comprising the filler and binder are separated. The row of small pores is believed to be pores formed by volatilization of binder existing between particles.

In the OM image, HR2-G showed particle voids of approximately 50 µm in diameter, with clusters of small pores. On the other hand, C-HR2-G had significantly fewer particle voids and pores, and sizes were also smaller than those for HR2-G. For C-HR2-G, stronger interparticle bonding due to curing contributed to smaller and fewer particle voids being produced during grinding. This phenomenon was further confirmed by SEM observation.

In the SEM ×500 image, HR2-G shows pores aligned along the particle boundaries, while C-HR2-G has pores existing independently without alignment. The microstructural observations showed that cured graphite blocks had already released gases at low temperatures (150 °C curing).

**Figure 9:**
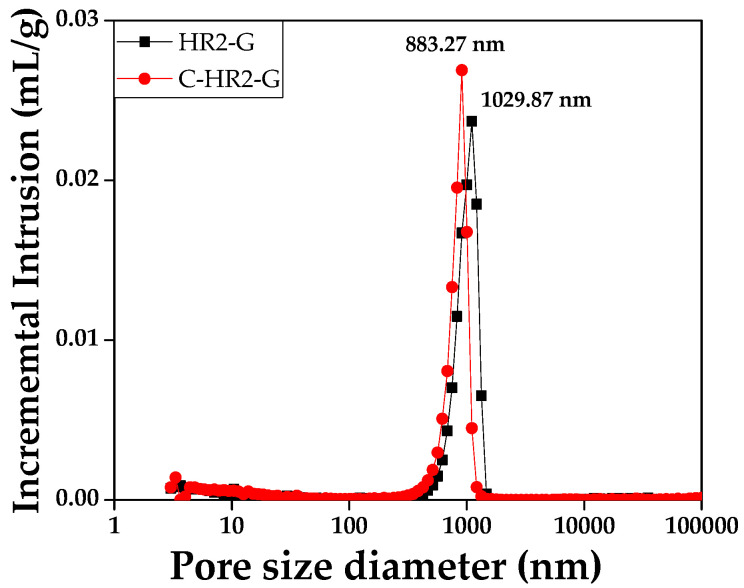
Incremental intrusion in range of pore size diameter 3 to 360,000 nm.

Figure 9 shows the incremental intrusion-median pore size graphs of HR2-G and C-HR2-G. Median pore diameter means the median value of a pore expressed as a cylindrical volume (the ranges were 175.62 to 204.77 in psi and 0.062 to 0.065 in mL/g). The median values of HR2-G and C-HR2-G were 1029.87 and 883.27 nm, respectively, which were inconsistent with the microstructure analysis.

#### 3.2.4. Oxidation Resistance Analysis

**Figure 10:**
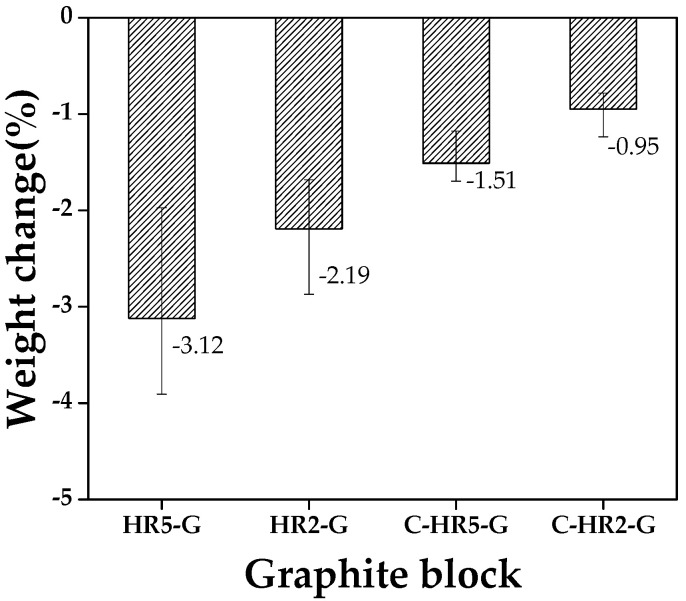
Weight change of graphite blocks before and after oxidation in relation to curing and carbonization conditions.

Figure 10 is a graph of weight changes of graphite blocks before and after oxidation in relation to curing and carbonization conditions. C-HR2-G had the lowest oxidation rate, with a weight loss of approximately 0.95%. This is consistent with the bulk density and porosity presented earlier. Table 3 shows the properties of the graphite blocks analyzed in this study, including the weight loss percentage measured after the oxidation test.

**Figure 11:**
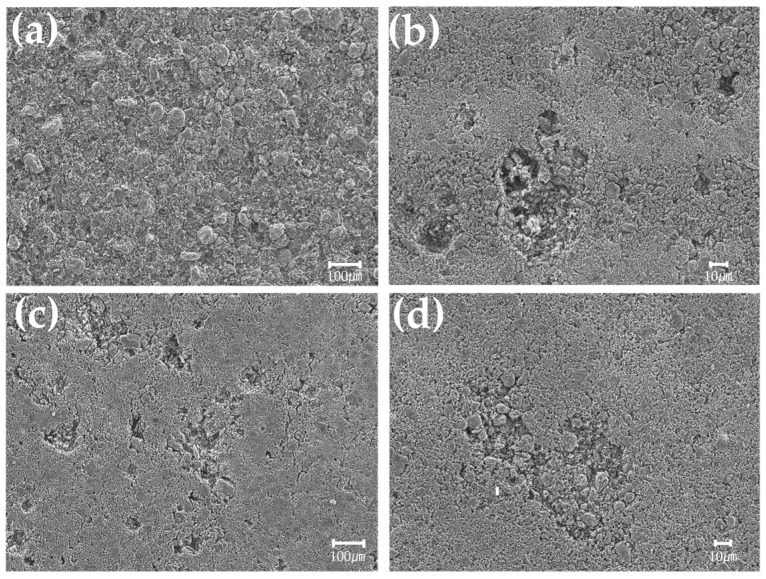
Microstructures of graphite blocks after oxidation in relation to curing and carbonization conditions: (**a**) HR5-G (×100), (**b**) HR2-G (×100), (**c**) C-HR5-G (×100), and (**d**) C-HR2-G (×100).

Figure 11 shows the surface observation results of graphite blocks after oxidation reactions. In Figure 11a, we can see that coke particles have fallen from the surface of HR5-G. HR2-G, which has a slower heating rate, showed partial separation of coke particles. The cured samples C-HR5-G and C-HR2-G also showed partial separation of coke particles.

Xiaowei, et al., explained that Blanchard reported that the oxidation of artificial graphite occurs by gas diffusing through pores in the temperature range of 600–900 °C [38,39]. Cured graphite blocks have low porosity and relatively fewer large pores, leading to excellent oxidation resistance. This is consistent with the microstructural results presented above. As such, we can conclude that the pore size of the graphite blocks plays an important role in oxidation.

#### 3.2.5. Physical Properties Change after Oxidation

In order to confirm the effect of the curing process on the improvement of oxidation resistance of graphite blocks, the change in physical properties before and after oxidation of HR2-G and C-HR2-G was confirmed (Table 4).

After the oxidation of HR2-G and C-HR2-G, the bulk density decreased by 1.30% and 0.35%, respectively, and the porosity increased by 11.60% and 4.43%, respectively. D. Chen, et al., reported the oxidation behavior of nuclear graphite. It has been reported that the oxidation reaction of graphite block allows more oxidizing gas to permeate as the porosity increases, and as the oxidation proceeds, the reaction surface of graphite gradually widens, resulting in a greater increase in porosity after oxidation [40]. Therefore, it was considered that C-HR2-G introduced in the curing process had a smaller reaction area through which gas could permeate in the oxidation reaction and thus had smaller porosity after oxidation.

The increase in porosity after oxidation, with or without the curing process, had a great effect on the electrical resistivity and flexural strength. After oxidation, the electrical resistivity of HR2-G and C-HR2-G increased by 20.25% and 9.09%, respectively, and the flexural strength decreased by 50.42% and 19.70%, respectively. Irregular surfaces formed by oxidation can decrease mechanical strength due to stress concentration effects [41]. As shown in Figure 10, since the surface of HR2-G without curing is rougher, it is thought that the bending strength is lowered due to stress concentration.

## 4. Conclusions

In this study, graphite blocks with controlled pore size were fabricated by introducing a curing process and varying the heating rate during carbonization. As a result, the following conclusions were obtained.

The methylene index was calculated from the FTIR; it was confirmed that the cross-linking of cured phenolic resin was maximized at 150 °C.

The gas release was lower at high temperatures (around 500 °C) during carbonization of 150 °C-cured phenol than it was in the case of non-cured phenol because the cured phenol resin had already released gases at low temperatures (150 °C) during curing. SEM observation showed that, as a result, large pores did not form in the cured and carbonized phenol.

Among the graphite blocks subject to various curing and carbonization conditions, C-HR2-G showed the best properties, with a bulk density of 1.707 g/cm^3^, porosity of 15.84%, electrical resistance of 23.1 μΩm, and flexural strength of 26.9 MPa. C-HR5-G also had better properties than those of HR2-G. This indicates that controlling the pore size by introducing curing is more effective at improving the physical properties than varying the heating rate.

C-HR2-G showed the best oxidation resistance because the curing process inhibited the formation of large pores in the graphite block. After oxidation, the physical properties of HR2-G and C-HR2-G deteriorated, with C-HR2-G exhibiting less physical property loss. The introduction of a curing process enables pore size control in graphite blocks and improves oxidation resistance.

The results of this study are expected to be applicable to graphite refractories that are sensitive to oxidation. The introduction of the curing process has the advantage of being able to room temperature molding and reduce process time. However, the limitation of still lower electrical properties compared to pitch is a problem to be overcome.

## Figures and Tables

**Table 1:** Naming of graphite blocks fabricated in this study.

Samples	Curing	Heating Rate during Carbonization (°C/min)	Graphitization
HR5-G	-	5	O
HR2-G	-	2	O
C-HR5-G	O	5	O
C-HR2-G	O	2	O

**Table 2:** Observed wave number (cm^−1^) and functional groups of FTIR spectra [34,35].

Observed Wave Number (cm^−1^)	Functional Group
3272~3364	OH stretch
2923~2916	aliphatic CH asymmetric stretch
2849	aliphatic CH symmetric stretch
1594~1610	C=C aromatic ring
1508~1509	C=C aromatic ring
1438~1472	aliphatic CH_2_ scissor bending
1369	phenolic OH in-plane deformation
1206~1234	alkyl-phenol C-O stretch
1095~1099	aromatic CH in-plane deformation
1005~1050	C-O stretch

**Table 3:** Physical properties of graphite blocks in relation to curing and carbonization conditions.

Samples	Bulk Density (g/cm^3^)	Porosity (%)	Electrical Resistivity (μΩm)	Flexural Strength (MPa)	Weight Change after Oxidation (%)
HR5-G	1.682	18.8	24.6	19.3	−3.12
HR2-G	1.696	18.1	24.2	23.8	−2.19
C-HR5-G	1.699	17.4	23.6	26.2	−1.51
C-HR2-G	1.707	15.8	23.1	26.9	−0.95

**Table 4:** Physical properties of graphite blocks in relation to curing and carbonizing conditions.

Samples	Oxidation	Bulk Density (g/cm^3^)	Porosity (%)	Electrical Resistivity (μΩm)	Flexural Strength (MPa)
HR2-G	Before	1.696	18.1	24.2	23.8
	After	1.674	20.2	29.1	11.8
	Δ%	−1.30	+11.60	+20.25	−50.42
C-HR2-G	Before	1.707	15.8	23.1	26.9
	After	1.701	16.5	25.2	21.6
	Δ%	−0.35	+4.43	+9.09	−19.70

## Data Availability

Not applicable.

## References

[1] Lee S.M., Kang D.S., Kim W.S., Roh J.S. (2014). Fabrication of Isotropic Bulk Graphite Using Artificial Graphite Scrap. Carbon Lett..

[2] Lee S.M., Kang D.S., Roh J.S. (2015). Bulk Graphite: Materials and Manufacturing Process. Carbon Lett..

[3] An D.H., Kim K.H., Lim C.H., Lee Y.S. (2021). Effect of Kneading and Carbonization Temperature on the Structure of the Carbon Block for Thermally Conductive Bulk Graphites. Carbon Lett..

[4] Knop A., Plato L.A. (1985). Phenolic Resins: Chemistry, Applications and Performance Future Directions.

[5] Irie S., James R. (2010). Phenolic Resins: A Century of Progress.

[6] Yang Z., Liu B., Zhao H., Li J., Guo X., Zhang D., Liu Z. (2023). Pyrolysis Mechanism of Composite Binder Composed of Coal Tar Pitch and Phenolic Resin for Carbon Materials. J. Anal. Appl. Pyrolysis.

[7] Propp W.A. (1998). Graphite Oxidation Thermodynamics/Reactions.

[8] Choi W.K., Kim B.J., Chi S.H., Park S.J. (2009). Nuclear graphites (I): Oxidation behaviors. Carbon Lett..

[9] Conejo L.S., Costa M.L., Oishi S.S., Botelho E.C. (2017). Degradation Behavior of Carbon Nanotubes/Phenol-Furfuryl Alcohol Multifunctional Composites with Aerospace Application. Mater. Res. Express.

[10] Khezrabadi M.N., Javadpour J., Rezaie H.R., Naghizadeh R. (2006). The Effect of Additives on the Properties and Microstructures of Al2O3-C Refractories. J. Mater. Sci..

[11] Dutta S., Das P., Das A., Mukhopadhyay S. (2014). Significant Improvement of Refractoriness of Al_2_O_3_–C Castables Containing Calcium Aluminate Nano-Coatings on Graphite. Ceram. Int..

[12] Zhang S., Lee W.E. (2003). Improving the Water-Wettability and Oxidation Resistance of Graphite Using Al_2_O_3_/SiO_2_ Sol-Gel Coatings. J. Eur. Ceram. Soc..

[13] Zhao H., Xie D., Zhang S., Du F. (2020). Study on Improving the High-Temperature Oxidation Resistance of Pyrolytic Carbons of Phenolic Resin Binder by in-Situ Formation of Carbon Nanotubes. React. Funct. Polym..

[14] Kang D.S., Kim B.J., Lee K.J., Kim S.H., Lee S.W., Roh J.S. (2013). Developing hollow carbon balls by oxidation of carbon blacks. Carbon Lett..

[15] Shin Y.W. (2005). A study of mechanical properties on high density graphite products with expanded graphite(1). J. Power Syst. Eng..

[16] Matzinos P.D., Patrick J.W., Walker A. (1996). Coal-tar pitch as a matrix precursor for 2-DC/C composites. Carbon.

[17] Youm H.N., Kim K.J., Lee J.M., Chung Y.J. (1993). Effects of impregnation on the manufacture of high density carbon materials. Yoop Hakhoechi.

[18] Zhao Y., Li X., Jia X., Gao S. (2019). Why and How to Tailor the Vertical Coordinate of Pore Size Distribution to Construct ORR-Active Carbon Materials?. Nano Energy.

[19] Lee S.M., Lee S.H., Roh J.S. (2021). The Pore-Filling Effect of Bulk Graphite According to Viscosity of Impregnant. Korean J. Mater. Res..

[20] Lee S.H., Kim J.H., Kim W.S., Roh J.S. (2022). The Effect of the Heating Rate during Carbonization on the Porosity, Strength, and Electrical Resistivity of Graphite Blocks Using Phenolic Resin as a Binder. Materials.

[21] Kim J.H., Park S.M., Lim Y.S., Park H.S., Kim M.S. (2002). Effect of KOH addition on pore structure of glassy carbon prepared by polymerization of phenolic resin. Polym. Korea.

[22] Maleki H., Holland L.R., Jenkins G.M., Zimmerman R.L. (1997). Determining the shortest production time for glassy carbon ware. Carbon.

[23] Jurkiewicz K., Pawlyta M., Zygadło D., Chrobak D., Duber S., Wrzalik R., Burian A. (2018). Evolution of glassy carbon under heat treatment: Correlation structure–mechanical properties. J. Mater. Sci..

[24] Walker P.L. (1987). Chemistry and Physics of Carbon: A Series of Advances.

[25] Pesin L.A. (2002). Review Structure and properties of glass-like carbon. J. Mater. Sci..

[26] Hajihosseini S., Nasirizadeh N., Hejazi M.S., Yaghmaei P. (2016). A Sensitive DNA Biosensor Fabricated from Gold Nanoparticles and Graphene Oxide on a Glassy Carbon Electrode. Mater. Sci. Eng. C.

[27] Tadyszak K., Litowczenko J., Majchrzycki Ł., Jeżowski P., Załęski K., Scheibe B. (2020). Sucrose Based Cellular Glassy Carbon for Biological Applications. Mater. Chem. Phys..

[28] Acuña N.T., Güiza A.V., Córdoba T.E. (2020). Reticulated Vitreous Carbon Foams from Sucrose: Promising Materials for Bone Tissue Engineering Applications. Macromol. Res..

[29] Xu Y., Guo L., Zhang H., Zhai H., Ren H. (2019). Research Status, Industrial Application Demand and Prospects of Phenolic Resin. RSC Adv..

[30] Chow S. (1972). Thermal Analysis of Liquid Phenol-Formaldehyde Resin Curing. Holzforschung.

[31] Yoon S.B., Kim J.W., Cho D.H. (2006). Thermal Stability and Cure Behavior of Waterborne Phenol-Formaldehyde Resin. J. Adhes. Interface.

[32] Hu H., Wang W., Jiang L., Liu L., Zhang Y., Yang Y., Wang J. (2022). Curing Mechanism of Resole Phenolic Resin Based on Variable Temperature FTIR Spectra and Thermogravimetry-Mass Spectrometry. Polym. Polym. Compos..

[33] Kejian J., Werhua D., Yusheng Y. (2002). Determination of the hydroxymethyl index for phenolic resin by infrared spectroscopy. Chem. Anal. Meter..

[34] Ricci A., Oleja K.J., Parpinello G.P., Killmartin P.A., Versari A. (2015). Application of furrier transform infrared (FTIR) spectroscopy in the characterization of tannins. Appl. Spectrosc. Rev..

[35] Cao Z., Wang Z., Shang Z., Zhao J. (2017). Classification and identification of Rhodobryum roseum Limpr. And its adulterants based on furrier-transform infrared spectroscopy (FTIR) and chemometrics. PLoS ONE.

[36] Chang C., Tackett J.R. (1991). Characterization of phenolic resins with thermogravimetry-mass spectrometry. Thermochim. Acta.

[37] Oshida K., Ekinaga N., Endo M., Inagaki M. (1996). Pore Analysis of Isotropic Graphite Using Image Processing of Optical Micrographs. TANSO.

[38] Xiaowei L., Jean C.R., Suyuan Y. (2004). Effect of Temperature on Graphite Oxidation Behavior. Nucl. Eng. Des..

[39] Blanchard A. (2000). The thermal oxidation of graphite-Irradiation damage in graphite due to fast neutrons in fission and fusion systems. IAEA-TECDOC.

[40] Chen D., Li Z., Miao W., Zhang Z. (2012). Effects of porosity and temperature on oxidation behavior in air of selected nuclear graphites. Mater. Trans..

[41] Matthews A.C., Kane J.J., Swank W.D., Windes W.E. (2021). Nuclear graphite strength degradation under varying oxidizing conditions. Nucl. Eng. Des..

